# Plasma brain-derived neurotrophic factor concentration is a predictor of chronic kidney disease in patients with cardiovascular risk factors – Hyogo Sleep Cardio-Autonomic Atherosclerosis study

**DOI:** 10.1371/journal.pone.0178686

**Published:** 2017-06-02

**Authors:** Masafumi Kurajoh, Manabu Kadoya, Akiko Morimoto, Akio Miyoshi, Akinori Kanzaki, Miki Kakutani-Hatayama, Kae Hamamoto, Takuhito Shoji, Yuji Moriwaki, Tetsuya Yamamoto, Masaaki Inaba, Mitsuyoshi Namba, Hidenori Koyama

**Affiliations:** 1 Department of Internal Medicine, Division of Diabetes, Endocrinology and Metabolism, Hyogo College of Medicine, Nishinomiya, Hyogo, Japan; 2 Department of Metabolism, Endocrinology, and Molecular Medicine, Osaka City University Graduate School of Medicine, Osaka, Japan; The University of Tokyo, JAPAN

## Abstract

**Background:**

Brain-derived neurotrophic factor (BDNF) has been shown to have protective effects against cardiovascular diseases and death through neural and non-neural pathways via tropomyosin-related kinase B signaling. However, it is not known whether plasma BDNF concentration is a predictor of chronic kidney disease (CKD).

**Design:**

This study was conducted as a prospective cohort study as part of the Hyogo Sleep Cardio-Autonomic Atherosclerosis.

**Methods:**

We measured plasma BDNF concentration in 324 patients without CKD, defined as an estimated glomerular filtration rate (eGFR) less than 60 ml/min/1.73m^2^, and with cardiovascular risk factors. As potential confounders, sleep condition, nocturnal hypertension, and autonomic function were quantitatively examined. The patients were followed for a median 37 months (range 2–59 months) and occurrence of CKD was noted.

**Results:**

Plasma BDNF concentration was significantly and independently associated with CKD development, which occurred in 38 patients (11.7%). Kaplan-Meier analysis revealed that patients with reduced plasma BDNF concentration exhibited a significantly (p = 0.029) greater number of CKD events as compared to those with a higher concentration. Moreover, comparisons of key subgroups showed that the risk of CKD in association with low plasma BDNF concentration was more prominent in patients with a greater reduction of nocturnal systolic blood pressure, better movement index, higher standard deviations of the NN(RR) interval or average NN(RR) interval for each 5-minute period, and without past cardiovascular disease events, smoking habit, or albuminuria.

**Conclusions:**

Plasma BDNF concentration is an independent predictor for development of CKD in patients with cardiovascular risk factors.

## Introduction

Chronic kidney disease (CKD) has become a major public health problem and its prevalence is increasing worldwide [1], while individuals with cardiovascular risk factors, such as diabetes, hypertension, dyslipidemia, obesity, and smoking habit, are known to be at greater risk for its development [2]. In addition, autonomic dysfunction, nocturnal hypertension, and sleep disturbance are frequently observed in patients with such risk factors [3–5] and those have been shown to be predictors of renal function decline [6–8], though the precise mechanism remains unclear. Reduced estimated glomerular filtration rate (eGFR) (<60 ml/min/1.73m^2^) is considered to be a risk factor for end-stage renal failure (ESRD), hospitalization, cardiovascular events, and death [9], thus elucidation of predictors for development of CKD is essential for treatment of patients with cardiovascular risk factors.

Brain-derived neurotrophic factor (BDNF), a member of the neurotrophin family of growth factors, was originally discovered in brain tissues [10]. It binds to tropomyosin-related kinase B (TrkB) [11] and regulates the survival, growth, and maintenance of neurons [12, 13]. BDNF has also been detected in systemic circulation [14], while TrkB expression has been found in a variety of non-neuronal tissues, including those of the heart, blood vessels, lungs, liver, and kidneys [15, 16]. Previous cross-sectional studies have shown that plasma BDNF concentration is decreased in patients with type 2 diabetes [17] and acute coronary syndrome [18], and it has also been reported to be correlated with obesity, blood pressure (BP), and lipid metabolism [19, 20]. In our previous investigations, we found that plasma BDNF concentration is associated with autonomic function and nocturnal BP fluctuation [4, 20], while Bachmann showed that BDNF polymorphism affects sleep function in healthy subjects [21]. Moreover, prospective studies have revealed that lower plasma BDNF concentration predicts higher all-cause mortality and coronary events [22, 23]. Thus, BDNF appears to have roles in energy metabolism, autonomic function, nocturnal hypertension, and sleep disturbance, and may also be protective against cardiovascular diseases and death through both neural [24] and non-neural [25] pathways via TrkB signaling.

Recently, BDNF administration was shown to repair renal damage via TrkB signaling in glomerular podocytes in rodents [26]. However, a relationship between plasma BDNF concentration and renal outcome in human subjects has not been shown. In the present study, we examined the relationship between plasma BDNF concentration and future development of CKD in patients with cardiovascular risk factors as part of the Hyogo Sleep Cardio-Autonomic Atherosclerosis (HSCAA) cohort study.

## Materials and methods

### 1. Study design and participants

The HSCAA study was conducted with patients with 1 or more cardiovascular risk factors (obesity, smoking habit, presence of cardiovascular event history, hypertension, dyslipidemia, diabetes mellitus, CKD) and designed to examine the impact of sleep, cardiac autonomic dysfunction, and subclinical atherosclerosis on cardiovascular events. Plasma BDNF concentrations were measured in those patients, who also had cardiac autonomic function, nocturnal BP, and sleep function fully measured. Among 428 patients with plasma BDNF concentrations determined, those with CKD defined as eGFR less than 60 ml/min/1.73m^2^ [27] (n = 59), and those never followed (n = 45) were excluded, thus 324 were analyzed in the present study. All variables, except for serum creatinine and eGFR, were measured only at the baseline. All patients registered between November 2010 and July 2013 agreed to participate in the present study by providing written informed consent, and the protocol was approved by the Ethics Committee of Hyogo College of Medicine (approval No. 948).

### 2. Clinical assessment

Details of the cohort have been previously reported [5]. Serum creatinine concentration was measured using an enzymatic method. eGFR was calculated using a modified MDRD equation for Japanese subjects, as previously described [28], and 24-hour urinary albumin excretion (UAE) was measured by immunoturbidimetry [3], with albuminuria defined as UAE equal to or greater than 30 mg/day.

### 3. Sleep disorder and sleep conditions

Apnea hypopnea index (AHI), sleep duration, sleep efficiency (percentage of time scored as sleep), and movement index (percentage of total number of mobile 60-s epochs with movement during sleep time) were determined using an Apnomonitor or Actigraph, as previously described in detail [4, 20].

### 4. Ambulatory blood pressure monitoring (ABPM)

Daytime and nighttime mean systolic blood pressure (SBP), and diastolic blood pressure values were measured using ABPM. Nocturnal SBP fall (%) was calculated as 100 x [1-sleep SBP/awake SBP ratio], as previously described in detail [20].

### 5. Cardiac autonomic nervous function

To evaluate cardiac autonomic function, the standard deviations of the NN(RR) interval (SDNN) and average NN(RR) interval for each 5-minute (SDANN5) period were calculated using an Active Tracer, as previously described in detail [3, 29].

### 6. Plasma BDNF concentration

Plasma BDNF concentrations were measured with an enzyme-linked immunosorbent assay, as previously described in detail [20].

### 7. Outcome data collection

The subjects were followed until December 2015, for a median follow-up period of 37 months (range 2–59 months). Essentially, serum creatinine concentrations were measured every 3 months and eGFR was calculated at each measurement time point. Development of CKD was defined as a decline in eGFR to less than 60 ml/min/1.73m^2^.

### 8. Statistical analyses

Values for BDNF and heart rate variability (HRV) parameters (SDNN and SDANN5) were natural logarithm-transformed (ln) to achieve a normal distribution. Pearson’s correlation coefficient was used to determine correlations between eGFR and plasma BDNF concentration. A non-repeated t-test (continuous variables with normal distribution) and a chi-square test (categorical variables) were used to compare variables between groups. The follow-up period is expressed as the median (limit of observed values). Patients were arbitrarily divided into 2 groups according to the median plasma BDNF concentration (higher, ≥2168 pg/ml; lower, <2168 pg/ml). The rates of incidence of development of CKD were compared using Kaplan-Meier analysis and log-rank test results. Prognostic variables for development of CKD were examined using a univariate or multivariable Cox proportional hazards regression model. The core of the analysis was based on a battery of Cox regressions. All statistical analyses were performed using the Statistical Package for the Social Sciences software (PASW Statistics version 18.0). All reported p values are 2-tailed and considered to be statistically significant at <0.05.

## Results

### Baseline clinical characteristics

The characteristics of the 324 subjects are shown in Table 1. Mean eGFR and plasma ln BDNF concentration were 86.8±19.9 ml/min/1.73m^2^ and 7.7±0.8 pg/ml, respectively. There was no significant correlation between eGFR and plasma BDNF concentration (r = 0.031, p = 0.574), and no significant difference in regard to eGFR between the higher and lower BDNF group (85.8±17.8 vs. 87.6±22.0 ml/min/1.73m^2^, p = 0.406).

**Table 1:** Clinical characteristics of subjects (n = 324).

Age, years	57.9±13.0
Male, n (%)	176 (54.3)
Body mass index, kg/m^2^	24.4±4.7
Diabetes mellitus, n (%)	122 (37.7)
Hypertension, n (%)	199 (61.4)
Dyslipidemia, n (%)	183 (56.5)
Past CVD events, n (%)	40 (12.3)
Medical treatments	
Ca antagonist, n (%)	128 (39.5)
ACE or ARB, n (%)	78 (24.1)
β or αβ blocker, n (%)	35 (10.8)
Diuretics, n (%)	15 (4.6)
Antiplatelet agent, n (%)	33 (10.2)
Antidepressant agent, n (%)	2 (0.6)
Current smoker, n (%)	84 (25.9)
eGFR, ml/min/1.73 m^2^	86.8±19.9
Albuminuria, n (%)	40 (12.3)
AHI	9.1±9.6
Sleep duration, min	379.6±95.5
Sleep Efficiency, %	90.3±7.3
Movement index, %	38.2±15.0
Nocturnal SBP fall, %	8.5±7.7
ln (SDNN), msec	4.78±0.28
ln (SDANN5), msec	4.66±0.31
ln (BDNF), pg/ml	7.7±0.8

Data are presented as the mean ± standard deviation or n (%) for dichotomous variables. HRV parameters (SDNN, SDANN5) and BDNF concentration were natural logarithm-transformed (ln) to achieve a normal distribution. Abbreviations: CVD, cardiovascular disease; ACE, angiotensin converting enzyme; ARB, angiotensin II receptor blocker; eGFR, estimated glomerular filtration rate; AHI, apnea hypopnea index; SBP, systolic blood pressure; SDNN, standard deviation of NN(RR) interval; SDANN5, standard deviation of average NN(RR) interval for each 5-min period; BDNF, brain-derived neurotrophic factor

### Association between plasma BDNF concentration and development of CKD

**Fig 1:**
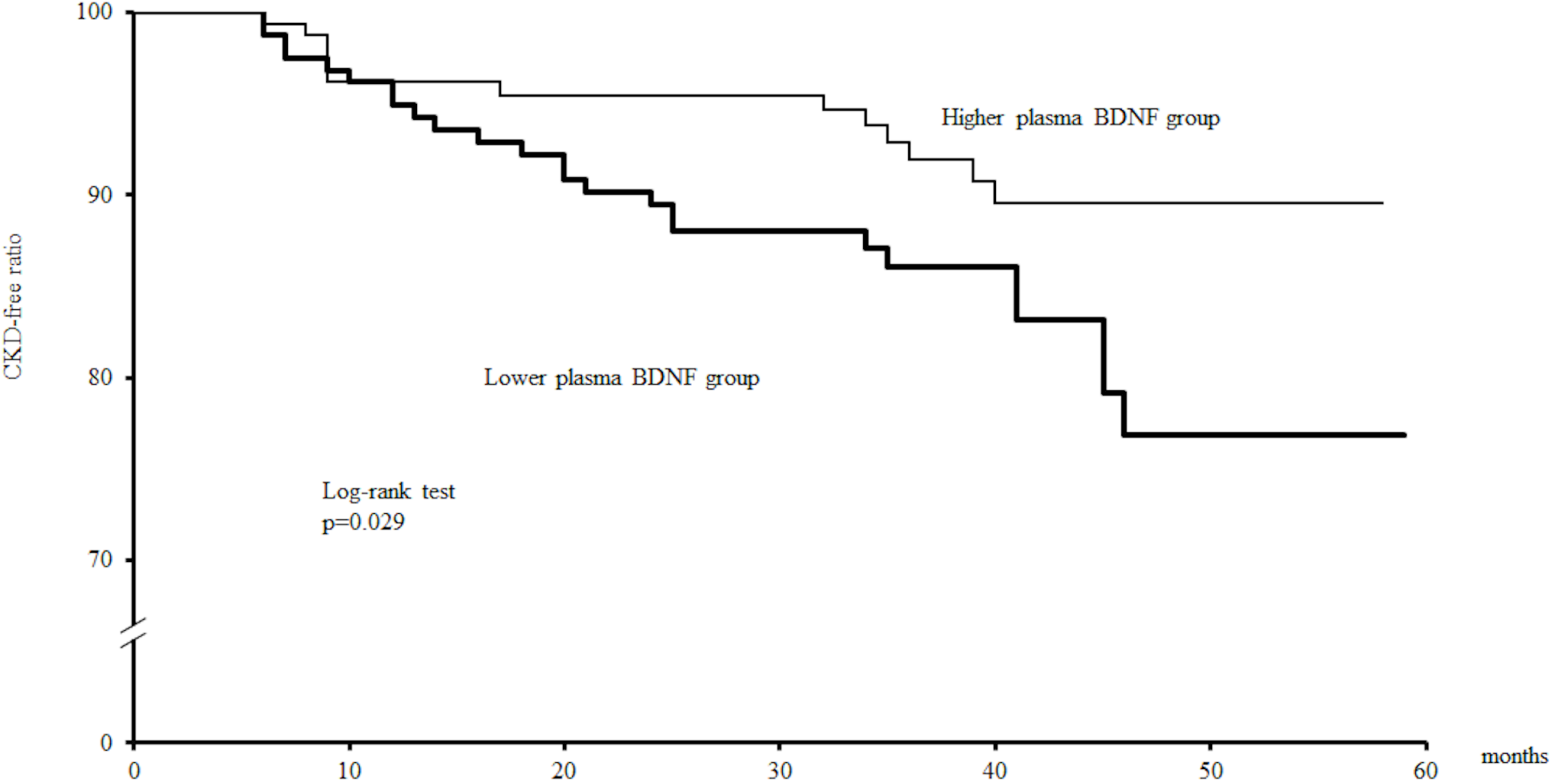
Findings of Kaplan-Meier analysis of association between plasma BDNF and CKD development. The patients were divided into 2 groups according to the median plasma BDNF level (≥2168 pg/ml, the median, and <2168 pg/ml). Probability value was analyzed using a log-rank test.

Of the 324 patients analyzed, 38 (11.7%) developed CKD during the follow-up period. Univariate Cox proportional analysis showed that plasma BDNF concentration was inversely associated with CKD development (Table 2). In addition, Kaplan-Meier analysis also indicated that patients with a lower plasma BDNF concentration had a significantly increased risk for development of CKD as compared to those with a higher plasma BDNF concentration (Fig 1).

**Table 2:** Univariate Cox proportional analysis of factors associated with CKD development.

	HR	95% CI	p
Age	1.052	1.020–1.084	0.001
Gender (female = 0, male = 1)	1.180	0.620–2.247	0.614
Body mass index	0.981	0.911–1.056	0.612
Diabetes mellitus (absence = 0, presence = 1)	1.423	0.750–2.699	0.280
Hypertension (absence = 0, presence = 1)	2.185	1.034–4.616	0.041
Dyslipidemia (absence = 0, presence = 1)	1.870	0.927–3.771	0.080
Past CVD events (absence = 0, presence = 1)	0.626	0.192–2.037	0.437
Ca antagonist (absence = 0, presence = 1)	1.604	0.849–3.030	0.145
ACE or ARB (absence = 0, presence = 1)	1.035	0.490–2.188	0.927
β or αβ blocker (absence = 0, presence = 1)	2.403	1.054–5.477	0.037
Diuretics (absence = 0, presence = 1)	1.978	0.607–6.439	0.257
Antiplatelet agent (absence = 0, presence = 1)	0.997	0.354–2.811	0.996
Current smoking (absence = 0, presence = 1)	1.095	0.532–2.255	0.806
eGFR	0.925	0.895–0.956	0.000
Albuminuria (absence = 0, presence = 1)	1.354	0.566–3.240	0.496
AHI	1.019	0.988–1.051	0.231
Sleep duration	0.999	0.996–1.002	0.451
Sleep efficiency	0.970	0.935–1.007	0.111
Movement index	1.015	0.995–1.034	0.137
Nocturnal SBP fall	1.013	0.971–1.057	0.553
ln (SDNN)	0.281	0.085–0.929	0.038
ln (SDANN5)	0.358	0.121–1.056	0.063
ln BDNF	0.661	0.452–0.965	0.032

Abbreviations: CVD, cardiovascular disease; eGFR, estimated glomerular filtration rate; SBP, systolic blood pressure; AHI, apnea hypopnea index; SDNN, standard deviation of NN(RR) interval; SDANN5, standard deviation of average NN(RR) interval for each 5-min period; BDNF, brain-derived neurotrophic factor

### Other clinical parameters associated with development of CKD

Older age, lower eGFR, lower SDNN, presence of hypertension, and use of a β or αβ blocker were significantly associated with development of CKD (Table 2), while lower SDANN5 and presence of dyslipidemia were borderline significant risks for its development. Gender, BMI, AHI, sleep duration, sleep efficiency, movement index, nocturnal SBP fall, presence of diabetes, past CVD events, smoking habit, and albuminuria, as well as use of Ca antagonist, angiotensin converting enzyme or angiotensin II receptor blocker, diuretics, and antiplatelet agent were not significantly associated with CKD development.

### Plasma BDNF concentration predicts development of CKD independent of other clinical factors

To further examine whether plasma BDNF concentration is independently associated with development of CKD, multivariate Cox proportional hazards analyses were performed (Table 3, S1 File). In basic model 1, which included BDNF, age, male gender, BMI, eGFR, presence of diabetes, hypertension, dyslipidemia, past CVD events, current smoking, and albuminuria as covariates, lower BDNF and eGFR [hazard ratio (HR), 0.927; 95% confidence interval (CI), 0.894–0.962; p<0.001] were significantly associated with development of CKD. When AHI, sleep duration, sleep efficiency, movement index, nocturnal SBP fall, SDNN, SDANN5, and medical treatment were sequentially added to model 1 (models 2–13), lower BDNF and eGFR remained as significantly high risk factors for development of CKD.

**Table 3:** Multivariate Cox proportional analysis of factors associated with CKD development.

Variables	HR (95% CI)	p
Model 1		
ln (BDNF)	0.604 (0.394–0.926)	0.021
Model 2		
ln (BDNF)	0.603 (0.394–0.923)	0.020
AHI	1.007 (0.968–1.047)	0.731
Model 3		
ln (BDNF)	0.599 (0.390–0.919)	0.019
Sleep duration	0.999 (0.995–1.002)	0.536
Model 4		
ln (BDNF)	0.607 (0.395–0.932)	0.023
Sleep efficiency	0.986 (0.948–1.025)	0.466
Model 5		
ln (BDNF)	0.603 (0.392–0.928)	0.021
Movement index	1.011 (0.990–1.033)	0.314
Model 6		
ln (BDNF)	0.597 (0.389–0.916)	0.018
Nocturnal SBP fall	1.017 (0.975–1.061)	0.433
Model 7		
ln (BDNF)	0.612 (0.396–0.944)	0.026
ln (SDNN)	0.407 (0.120–1.379)	0.149
Model 8		
ln (BDNF)	0.612 (0.398–0.942)	0.025
ln (SDANN5)	0.547 (0.190–1.577)	0.264
Model 9		
ln (BDNF)	0.602 (0.393–0.923)	0.020
Ca antagonist	0.923 (0.416–2.049)	0.844
Model 10		
ln (BDNF)	0.627 (0.408–0.964)	0.033
ACE or ARB	0.539 (0.235–1.234)	0.144
Model 11		
ln (BDNF)	0.622 (0.403–0.958)	0.031
β or αβ blocker	1.545 (0.652–3.662)	0.323
Model 12		
ln (BDNF)	0.587 (0.383–0.899)	0.014
Diuretics	2.810 (0.777–10.160)	0.115
Model 13		
ln (BDNF)	0.599 (0.390–0.920)	0.019
Antiplatelet agents	0.793 (0.258–2.442)	0.687

Model 1 included BDNF, age, male gender, BMI, eGFR, presence of diabetes, hypertension, dyslipidemia, past CVD events, current smoking, and albuminuria as covariates. In other models, AHI (Model 2), sleep duration (Model 3), sleep efficiency (Model 4), movement index (Model 5), nocturnal SBP fall (Model 6), SDNN (Model 7), SDANN5 (Model 8), use of Ca antagonist (Model 9), ACE or ARB (Model 10), β or αβ blocker (Model 11), diuretics (Model 12), or antiplatelet agents was added to model 1. Abbreviations: BDNF, brain-derived neurotrophic factor; AHI, apnea hypopnea index; SBP, systolic blood pressure; SDNN, standard deviation of NN(RR) interval; SDANN5, standard deviation of average NN(RR) interval for each 5-min period; ACE, angiotensin converting enzyme; ARB, angiotensin II receptor blocker; BMI, body mass index; eGFR, estimated glomerular filtration rate; CVD, cardiovascular disease

### Association of low plasma BDNF concentration with CKD development in specific key groups

**Fig 2:**
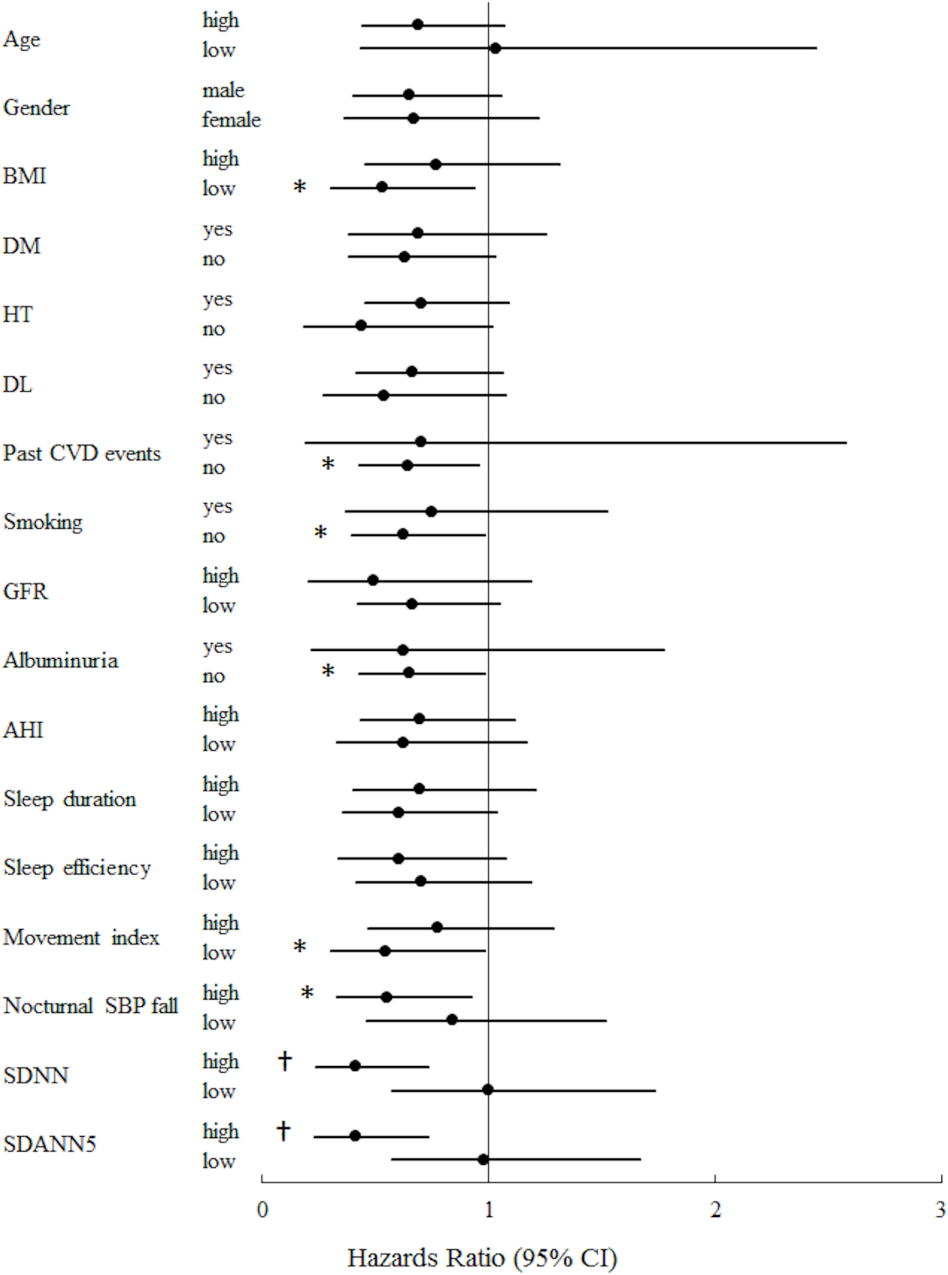
Comparisons of plasma BDNF level as a risk factor for CKD development in key subgroups (univariate cox proportional hazard analysis). We defined the higher and lower groups as greater than or equal to the median and less than the median, respectively. The median values for age, BMI, eGFR, AHI, sleep duration, sleep efficiency, movement index, nocturnal SBP fall, SDNN, and SDANND were 61 years, 23.75 kg/m^2^, 82 ml/min/1.73 m^2^, 5.75, 393.75 min, 92.07%, 36.10%, 9.09%, 117.05 msec, and 105.85 msec, respectively. *P <0.05; ^†^P <0.01. CI, confidence interval.

The association of BDNF with CKD development in specific key groups of subjects is shown in Fig 2. Of interest, the association of lower plasma BDNF concentration with CKD development was more prominent in patients with higher levels of nocturnal SBP fall, SDNN, and SDANN5, and lower movement index.

## Discussion

This is the first study to investigate the relationship between plasma BDNF concentration and development of CKD. Previously, BDNF was shown to modulate obesity, diabetes, hypertension, and dyslipidemia [19], each of which is a traditional risk factor for CKD [2]. The present findings showed that plasma BDNF concentration is also associated with CKD development, independent of other known risk factors.

### Association of autonomic function, nocturnal hypertension, and sleep disturbance with development of CKD–involvement of BDNF

Autonomic dysfunction, nocturnal hypertension, and sleep disturbance are frequently observed in patients with risk factors for CKD, such as obesity, diabetes, hypertension, and dyslipidemia [3–5], and it is important to note that those factors have also been shown to be predictors of decline in renal function [6–8]. The ARIC study, which included 13,241 adults, found that autonomic function, assessed by HRV, is associated with incidence of end-stage renal disease [6], which was confirmed by our results. Over-activity of sympathetic neurons is considered to lead to renal damage via direct and indirect mechanisms, such as nitric oxide and catecholamine metabolism [30]. Furthermore, the Jackson Heart Study and Nurses’ Health study showed that decreased nocturnal BP fall and short sleep duration are associated with a decline in renal function [7, 8]. Inconsistent with previous reports, we found in the present study that nocturnal BP fall and sleep function including sleep duration were not significantly associated with incidence of CKD. This discrepancy may be partly explained by differences between our study of patients with cardiovascular risk factors and the above mentioned studies of population-based participants or female nurses. Sleep disturbance is thought to cause increased sympathetic nervous system activity, decreased nocturnal BP fall, and impaired metabolic regulation [31], while nocturnal hypertension and decreased nocturnal BP fall are thought to be caused by increased nocturnal activity of sympathetic neurons, poor sleep quality, high sodium sensitivity, and a lower level of diurnal activity [32]. Thus, reciprocally affected autonomic dysfunction, blunted nocturnal BP fall, and sleep disturbance might contribute to renal damage in both additive and synergistic manners, though the precise mechanism remains unclear.

Recent studies including ours have demonstrated that BDNF is associated with autonomic function, nocturnal hypertension, and sleep disturbance. We have shown that plasma BDNF concentration is associated with autonomic function and nocturnal BP fluctuation, and also mutually associated with autonomic function and nocturnal BP fluctuations in patients with cardiovascular risk factors [4, 20]. Others showed that BDNF signaling had effects on sleep homeostasis in rats via synaptic potentiation of the cerebral cortex [33], while human subjects with the BDNF polymorphism have been revealed to have impaired sleep slow wave activity, reflecting synaptic plasticity [21]. In the present study, comparisons of the association of BDNF with CKD development in key subgroups showed that the impact of plasma BDNF was more prominent in patients with higher nocturnal SBP fall, SDNN, SDANN5, and better movement index. Notably, plasma BDNF concentration was significantly associated with cardiac autonomic function and nocturnal SBP fall, suggesting that these parameters are important confounders for the association between BDNF and CKD development. Interestingly, the association of lower plasma BDNF concentration with CKD development was more prominent in patients with lower movement index as a preclinical study in which low BDNF levels were observed showed a decreased spontaneous locomotor activity [34]. Thus, the predictive effect of BDNF on CKD development may be overwhelmed by impaired regulation of cardiac autonomic function, nocturnal BP, and sleep function.

### Potential mechanisms of association between plasma BDNF concentration and CKD development

In addition to neuronal tissues, the BDNF receptor TrkB is expressed in non-neuronal tissues including the kidneys [16]. TrkB-deficient mice showed a significant reduction in glomerular areas, increased numbers of extra-glomerular mesangial cells, and absence of the macula densa [35]. Thus, TrkB signalling is considered to be critical for development and maintenance of a normal kidney architecture [35, 36]. Recently, administration of BDNF was shown to increase the number and length of cultured podocyte processes, and also improved glomerular damage by repairing podocyte injury in a mouse model of focal segmental glomerulosclerosis [26]. Since TrkB is nearly exclusively expressed in podocytes in humans [26], BDNF may directly protect against podocyte injury under conditions such as obesity, diabetes, hypertension, and dyslipidemia, in addition to glomerular disease. With that background in mind, we consider that BDNF administration may be a novel approach to inhibit development of CKD in humans. On the other hand, asymmetrical dimethylarginine (ADMA), an endogenous inhibitor of nitric oxide synthase, might be other potential mechanism to explain the association between plasma BDNF concentration and CKD development. ADMA interferes with renal function via renal vasoconstriction and a high level in plasma was found to predict progression of renal disease to ESRD [37]. Importantly, administration of ADMA was shown to decrease BDNF levels in patients with CKD [34]. Thus, BDNF might be associated with CKD development via ADMA accumulation, though we did not determine its concentration in plasma in the present study.

### Limitations

This study has some limitations. First, we used reduced eGFR (less than 60 ml/min/1.73m^2^) as a clinical endpoint rather than albuminuria or ESRD. Also, urinary albumin level and protein-to-creatinine ratio were not fully determined. Furthermore, none of the participants developed ESRD during the study period, thus we were not able to analyze the effect of BDNF level on albuminuria or hard renal endpoints. Second, the number of study subjects was relatively small and the follow-up duration was relatively short, which might explain why important risk factors for CKD, such as diabetes and albuminuria, were not shown to be associated with CKD development in this study. Further longitudinal follow-up examinations of this cohort are needed to clarify the role of BDNF in development of CKD, albuminuria, and ESRD. However, to the best of our knowledge, no other study has explored the association between BDNF and renal outcome together with autonomic function, sleep quality, and nocturnal hypertension. Third, no formal testing of depression or neurocognitive disorder, which are closely associated with CKD and BDNF [34, 38], was performed, though medical treatment for depression was checked in the present study. Fourth, the cure fraction for CKD was not estimated, because we performed standard but not extended Cox proportional hazards analyses. Finally, the present study population consisted of nearly exclusively Japanese patients with cardiovascular risk factors, thus it is unclear whether these findings can generalized for other ethnic groups or subjects without cardiovascular risk factors.

## Conclusions

Our results showed that plasma BDNF concentration is an independent predictor for development of CKD in patients with cardiovascular risk factors.

## Supporting information

S1 Table Supporting data. (XLSX)

S1 File Multivariate Cox proportional analysis of factors associated with CKD development. (DOCX)
