# Supplementary material for: Plasma brain-derived neurotrophic factor concentration is a predictor of chronic kidney disease in patients with cardiovascular risk factors – Hyogo Sleep Cardio-Autonomic Atherosclerosis study
Source: PLoS One. 2017 Jun 2;12(6):e0178686. doi: 10.1371/journal.pone.0178686 (PMC5456118; doi:10.1371/journal.pone.0178686)
Supplement: S1 File — (DOCX) [file pone.0178686.s002.docx]

**Supplementary Table 1. Multivariate Cox proportional analysis of factors associated with CKD development – Model 1**

| Variables | HR (95% CI) | p |
| --- | --- | --- |
| Age | 1.018 (0.981-1.056) | 0.336 |
| Gender (female=0, male=1) | 0.895 (0.439-1.824) | 0.760 |
| Body mass index | 0.998 (0.907-1.098) | 0.974 |
| Diabetes mellitus (absence=0, presence =1) | 1.287 (0.617-2.684) | 0.501 |
| Hypertension (absence=0, presence =1) | 1.648 (0.720-3.776) | 0.237 |
| Dyslipidemia (absence=0, presence =1) | 1.159 (0.522-2.570) | 0.717 |
| Past CVD events (absence=0, presence =1) | 0.363 (0.108-1.222) | 0.102 |
| Current smoking (absence=0, presence =1) | 1.141 (0.537-2.425) | 0.732 |
| eGFR | 0.927 (0.894-0.962) | <0.001 |
| Albuminuria (absence=0, presence =1) | 1.285 (0.501-3.294) | 0.602 |
| BDNF (ln) | 0.604 (0.394-0.926) | 0.021 |

Abbreviations: CVD, cardiovascular disease; eGFR, estimated glomerular filtration rate; BDNF, brain-derived neurotrophic factor; ln, natural logarithm transformed

**Supplementary Table 2. Multivariate Cox proportional analysis of factors associated with CKD development – Model 2**

| Variables | HR (95% CI) | p |
| --- | --- | --- |
| Age | 1.017 (0.978-1.056) | 0.401 |
| Gender (female=0, male=1) | 0.890 (0.437-1.816) | 0.749 |
| Body mass index | 0.992 (0.894-1.100) | 0.875 |
| Diabetes mellitus (absence=0, presence =1) | 1.288 (0.618-2.686) | 0.500 |
| Hypertension (absence=0, presence =1) | 1.644 (0.717-3.773) | 0.240 |
| Dyslipidemia (absence=0, presence =1) | 1.168 (0.525-2.601) | 0.703 |
| Past CVD events (absence=0, presence =1) | 0.360 (0.107-1.213) | 0.099 |
| Current smoking (absence=0, presence =1) | 1.149 (0.540-2.445) | 0.719 |
| eGFR | 0.928 (0.895-0.962) | <0.001 |
| Albuminuria (absence=0, presence =1) | 1.314 (0.508-3.399) | 0.574 |
| BDNF (ln) | 0.603 (0.394-0.923) | 0.020 |
| AHI | 1.007 (0.968-1.047) | 0.731 |

Abbreviations: CVD, cardiovascular disease; eGFR, estimated glomerular filtration rate; BDNF, brain-derived neurotrophic factor; ln, natural logarithm transformed; AHI, apnea hypopnea index

**Supplementary Table 3. Multivariate Cox proportional analysis of factors associated with CKD development – Model 3**

| Variables | HR (95% CI) | p |
| --- | --- | --- |
| Age | 1.019 (0.983-1.058) | 0.306 |
| Gender (female=0, male=1) | 0.864 (0.420-1.776) | 0.691 |
| Body mass index | 0.999 (0.908-1.099) | 0.982 |
| Diabetes mellitus (absence=0, presence =1) | 1.260 (0.602-2.637) | 0.540 |
| Hypertension (absence=0, presence =1) | 1.613 (0.702-3.707) | 0.260 |
| Dyslipidemia (absence=0, presence =1) | 1.131 (0.509-2.514) | 0.763 |
| Past CVD events (absence=0, presence =1) | 0.360 (0.107-1.211) | 0.099 |
| Current smoking (absence=0, presence =1) | 1.149 (0.539-2.448) | 0.720 |
| eGFR | 0.928 (0.895-0.962) | <0.001 |
| Albuminuria (absence=0, presence =1) | 1.305 (0.509-3.347) | 0.580 |
| BDNF (ln) | 0.599 (0.390-0.919) | 0.019 |
| Sleep duration | 0.999 (0.995-1.002) | 0.536 |

Abbreviations: CVD, cardiovascular disease; eGFR, estimated glomerular filtration rate; BDNF, brain-derived neurotrophic factor; ln, natural logarithm transformed

**Supplementary Table 4. Multivariate Cox proportional analysis of factors associated with CKD development – Model 4**

| Variables | HR (95% CI) | p |
| --- | --- | --- |
| Age | 1.017 (0.980-1.055) | 0.369 |
| Gender (female=0, male=1) | 0.897 (0.442-1.822) | 0.764 |
| Body mass index | 0.992 (0.899-1.094) | 0.872 |
| Diabetes mellitus (absence=0, presence =1) | 1.287 (0.617-2.685) | 0.502 |
| Hypertension (absence=0, presence =1) | 1.683 (0.731-3.873) | 0.221 |
| Dyslipidemia (absence=0, presence =1) | 1.129 (0.505-2.523) | 0.768 |
| Past CVD events (absence=0, presence =1) | 0.371 (0.110-1.251) | 0.110 |
| Current smoking (absence=0, presence =1) | 1.157 (0.543-2.469) | 0.706 |
| eGFR | 0.928 (0.895-0.962) | <0.001 |
| Albuminuria (absence=0, presence =1) | 1.242 (0.484-3.192) | 0.652 |
| ln BDNF | 0.607 (0.395-0.932) | 0.023 |
| Sleep efficiency | 0.986 (0.948-1.025) | 0.466 |

Abbreviations: CVD, cardiovascular disease; eGFR, estimated glomerular filtration rate; BDNF, brain-derived neurotrophic factor

**Supplementary Table 5. Multivariate Cox proportional analysis of factors associated with CKD development – Model 5**

| Variables | HR (95% CI) | p |
| --- | --- | --- |
| Age | 1.015 (0.979-1.054) | 0.418 |
| Gender (female=0, male=1) | 0.862 (0.421-1.768) | 0.686 |
| Body mass index | 0.990 (0.896-1.093) | 0.838 |
| Diabetes mellitus (absence=0, presence =1) | 1.280 (0.614-2.667) | 0.511 |
| Hypertension (absence=0, presence =1) | 1.666 (0.720-3.852) | 0.233 |
| Dyslipidemia (absence=0, presence =1) | 1.140 (0.510-2.547) | 0.750 |
| Past CVD events (absence=0, presence =1) | 0.377 (0.112-1.267) | 0.115 |
| Current smoking (absence=0, presence =1) | 1.135 (0.533-2.420) | 0.743 |
| eGFR | 0.927 (0.895-0.961) | <0.001 |
| Albuminuria (absence=0, presence =1) | 1.171 (0.451-3.038) | 0.746 |
| BDNF (ln) | 0.603 (0.392-0.928) | 0.021 |
| Movement index | 1.011 (0.990-1.033) | 0.314 |

Abbreviations: CVD, cardiovascular disease; eGFR, estimated glomerular filtration rate; BDNF, brain-derived neurotrophic factor; ln, natural logarithm transformed

**Supplementary Table 6. Multivariate Cox proportional analysis of factors associated with CKD development – Model 6**

| Variables | HR (95% CI) | p |
| --- | --- | --- |
| Age | 1.019 (0.982-1.058) | 0.308 |
| Gender (female=0, male=1) | 0.887 (0.436-1.806) | 0.741 |
| Body mass index | 1.002 (0.910-1.102) | 0.970 |
| Diabetes mellitus (absence=0, presence =1) | 1.247 (0.596-2.609) | 0.558 |
| Hypertension (absence=0, presence =1) | 1.682 (0.734-3.853) | 0.219 |
| Dyslipidemia (absence=0, presence =1) | 1.139 (0.515-2.521) | 0.748 |
| Past CVD events (absence=0, presence =1) | 0.365 (0.108-1.232) | 0.104 |
| Current smoking (absence=0, presence =1) | 1.134 (0.533-2.411) | 0.745 |
| eGFR | 0.927 (0.894-0.961) | <0.001 |
| Albuminuria (absence=0, presence =1) | 1.314 (0.513-3.367) | 0.569 |
| ln BDNF | 0.597 (0.389-0.916) | 0.018 |
| Nocturnal SBP fall | 1.017 (0.975-1.061) | 0.433 |

Abbreviations: CVD, cardiovascular disease; eGFR, estimated glomerular filtration rate; BDNF, brain-derived neurotrophic factor; SBP, systolic blood pressure

**Supplementary Table 7. Multivariate Cox proportional analysis of factors associated with CKD development – Model 7**

| Variables | HR (95% CI) | p |
| --- | --- | --- |
| Age | 1.016 (0.978-1.055) | 0.421 |
| Gender (female=0, male=1) | 0.904 (0.441-1.852) | 0.782 |
| Body mass index | 0.991 (0.900-1.092) | 0.858 |
| Diabetes mellitus (absence=0, presence =1) | 1.399 (0.656-2.986) | 0.385 |
| Hypertension (absence=0, presence =1) | 1.664 (0.732-3.785) | 0.224 |
| Dyslipidemia (absence=0, presence =1) | 1.059 (0.478-2.345) | 0.888 |
| Past CVD events (absence=0, presence =1) | 0.361 (0.107-1.222) | 0.101 |
| Current smoking (absence=0, presence =1) | 1.148 (0.539-2.446) | 0.720 |
| eGFR | 0.927 (0.894-0.962) | <0.001 |
| Albuminuria (absence=0, presence =1) | 1.184 (0.452-3.102) | 0.731 |
| BDNF (ln) | 0.612 (0.396-0.944) | 0.026 |
| SDNN (ln) | 0.407 (0.120-1.379) | 0.149 |

Abbreviations: CVD, cardiovascular disease; eGFR, estimated glomerular filtration rate; BDNF, brain-derived neurotrophic factor; ln, natural logarithm transformed, SDNN, standard deviation of NN(RR) interval

**Supplementary Table 8. Multivariate Cox proportional analysis of factors associated with CKD development – Model 8**

| Variables | HR (95% CI) | p |
| --- | --- | --- |
| Age | 1.017 (0.979-1.055) | 0.391 |
| Gender (female=0, male=1) | 0.883 (0.432-1.807) | 0.734 |
| Body mass index | 0.994 (0.903-1.094) | 0.905 |
| Diabetes mellitus (absence=0, presence =1) | 1.368 (0.644-2.908) | 0.415 |
| Hypertension (absence=0, presence =1) | 1.668 (0.733-3.797) | 0.222 |
| Dyslipidemia (absence=0, presence =1) | 1.083 (0.487-2.407) | 0.845 |
| Past CVD events (absence=0, presence =1) | 0.363 (0.108-1.227) | 0.103 |
| Current smoking (absence=0, presence =1) | 1.163 (0.546-2.474) | 0.696 |
| eGFR | 0.927 (0.894-0.961) | <0.001 |
| Albuminuria (absence=0, presence =1) | 1.221 (0.470-3.173) | 0.682 |
| BDNF (ln) | 0.612 (0.398-0.942) | 0.025 |
| SDANN5 (ln) | 0.547 (0.190-1.577) | 0.264 |

Abbreviations: CVD, cardiovascular disease; eGFR, estimated glomerular filtration rate; BDNF, brain-derived neurotrophic factor; ln, natural logarithm transformed, SDANN5, standard deviation of average NN(RR) interval for each 5-minute period

**Supplementary Table 9. Multivariate Cox proportional analysis of factors associated with CKD development – Model 9**

| Variables | HR (95% CI) | p |
| --- | --- | --- |
| Age | 1.018 (0.982-1.057) | 0.329 |
| Gender (female=0, male=1) | 0.891 (0.437-1.818) | 0.752 |
| Body mass index | 0.999 (0.908-1.099) | 0.976 |
| Diabetes mellitus (absence=0, presence =1) | 1.274 (0.607-2.672) | 0.522 |
| Hypertension (absence=0, presence =1) | 1.733 (0.661-4.541) | 0.264 |
| Dyslipidemia (absence=0, presence =1) | 1.156 (0.521-2.568) | 0.722 |
| Past CVD events (absence=0, presence =1) | 0.361 (0.107-1.217) | 0.100 |
| Current smoking (absence=0, presence =1) | 1.155 (0.538-2.481) | 0.711 |
| eGFR | 0.927 (0.894-0.961) | <0.001 |
| Albuminuria (absence=0, presence =1) | 1.315 (0.498-3.472) | 0.580 |
| BDNF (ln) | 0.602 (0.393-0.923) | 0.020 |
| Ca antagonist | 0.923 (0.416-2.049) | 0.844 |

Abbreviations: CVD, cardiovascular disease; eGFR, estimated glomerular filtration rate; BDNF, brain-derived neurotrophic factor; ln, natural logarithm transformed

**Supplementary Table 10. Multivariate Cox proportional analysis of factors associated with CKD development – Model 10**

| Variables | HR (95% CI) | p |
| --- | --- | --- |
| Age | 1.021 (0.984-1.059) | 0.273 |
| Gender (female=0, male=1) | 0.920 (0.452-1.873) | 0.818 |
| Body mass index | 0.992 (0.899-1.095) | 0.879 |
| Diabetes mellitus (absence=0, presence =1) | 1.392 (0.662-2.926) | 0.383 |
| Hypertension (absence=0, presence =1) | 1.962 (0.843-4.568) | 0.118 |
| Dyslipidemia (absence=0, presence =1) | 1.130 (0.513-2.491) | 0.761 |
| Past CVD events (absence=0, presence =1) | 0.425 (0.125-1.445) | 0.171 |
| Current smoking (absence=0, presence =1) | 1.151 (0.540-2.453) | 0.716 |
| eGFR | 0.925 (0.892-0.960) | <0.001 |
| Albuminuria (absence=0, presence =1) | 1.361 (0.522-3.550) | 0.529 |
| BDNF (ln) | 0.627 (0.408-0.964) | 0.033 |
| ACE or ARB | 0.539 (0.235-1.234) | 0.144 |

Abbreviations: CVD, cardiovascular disease; eGFR, estimated glomerular filtration rate; BDNF, brain-derived neurotrophic factor; ln, natural logarithm transformed, ACE, angiotensin converting enzyme; ARB, angiotensin II receptor blocker

**Supplementary Table 11. Multivariate Cox proportional analysis of factors associated with CKD development – Model 11**

| Variables | HR (95% CI) | p |
| --- | --- | --- |
| Age | 1.018 (0.981-1.056) | 0.342 |
| Gender (female=0, male=1) | 0.920 (0.453-1.866) | 0.817 |
| Body mass index | 0.997 (0.908-1.095) | 0.947 |
| Diabetes mellitus (absence=0, presence =1) | 1.325 (0.632-2.777) | 0.456 |
| Hypertension (absence=0, presence =1) | 1.521 (0.657-3.524) | 0.328 |
| Dyslipidemia (absence=0, presence =1) | 1.166 (0.530-2.567) | 0.702 |
| Past CVD events (absence=0, presence =1) | 0.371 (0.111-1.247) | 0.109 |
| Current smoking (absence=0, presence =1) | 1.156 (0.543-2.457) | 0.707 |
| eGFR | 0.929 (0.896-0.963) | <0.001 |
| Albuminuria (absence=0, presence =1) | 1.268 (0.494-3.256) | 0.622 |
| BDNF (ln) | 0.622 (0.403-0.958) | 0.031 |
| β or αβ blocker | 1.545 (0.652-3.662) | 0.323 |

Abbreviations: CVD, cardiovascular disease; eGFR, estimated glomerular filtration rate; BDNF, brain-derived neurotrophic factor; ln, natural logarithm transformed

**Supplementary Table 12. Multivariate Cox proportional analysis of factors associated with CKD development – Model 12**

| Variables | HR (95% CI) | p |
| --- | --- | --- |
| Age | 1.018 (0.980-1.056) | 0.364 |
| Gender (female=0, male=1) | 0.921 (0.454-1.868) | 0.820 |
| Body mass index | 0.990 (0.899-1.091) | 0.842 |
| Diabetes mellitus (absence=0, presence =1) | 1.377 (0.660-2.874) | 0.394 |
| Hypertension (absence=0, presence =1) | 1.566 (0.676-3.627) | 0.296 |
| Dyslipidemia (absence=0, presence =1) | 1.182 (0.531-2.632) | 0.683 |
| Past CVD events (absence=0, presence =1) | 0.334 (0.098-1.135) | 0.079 |
| Current smoking (absence=0, presence =1) | 1.044 (0.484-2.251) | 0.912 |
| eGFR | 0.927 (0.895-0.961) | <0.001 |
| Albuminuria (absence=0, presence =1) | 1.203 (0.465-3.112) | 0.702 |
| BDNF (ln) | 0.587 (0.383-0.899) | 0.014 |
| Diuretics | 2.810 (0.777-10.160) | 0.115 |

Abbreviations: CVD, cardiovascular disease; eGFR, estimated glomerular filtration rate; BDNF, brain-derived neurotrophic factor; ln, natural logarithm transformed

**Supplementary Table 13. Multivariate Cox proportional analysis of factors associated with CKD development – Model 13**

| Variables | HR (95% CI) | p |
| --- | --- | --- |
| Age | 1.018 (0.981-1.056) | 0.342 |
| Gender (female=0, male=1) | 0.886 (0.434-1.810) | 0.740 |
| Body mass index | 0.995 (0.903-1.097) | 0.924 |
| Diabetes mellitus (absence=0, presence =1) | 1.304 (0.622-2.733) | 0.483 |
| Hypertension (absence=0, presence =1) | 1.662 (0.725-3.810) | 0.230 |
| Dyslipidemia (absence=0, presence =1) | 1.182 (0.531-2.632) | 0.683 |
| Past CVD events (absence=0, presence =1) | 0.395 (0.111-1.411) | 0.153 |
| Current smoking (absence=0, presence =1) | 1.121 (0.525-2.394) | 0.767 |
| eGFR | 0.928 (0.895-0.962) | <0.001 |
| Albuminuria (absence=0, presence =1) | 1.313 (0.510-3.384) | 0.573 |
| BDNF (ln) | 0.599 (0.390-0.920) | 0.019 |
| Antiplatelet agents | 0.793 (0.258-2.442) | 0.687 |

Abbreviations: CVD, cardiovascular disease; eGFR, estimated glomerular filtration rate; BDNF, brain-derived neurotrophic factor; ln, natural logarithm transformed
